# Data Description Technique-Based Islanding Classification for Single-Phase Grid-Connected Photovoltaic System

**DOI:** 10.3390/s20113320

**Published:** 2020-06-11

**Authors:** Ahteshamul Haque, Abdulaziz Alshareef, Asif Irshad Khan, Md Mottahir Alam, Varaha Satya Bharath Kurukuru, Kashif Irshad

**Affiliations:** 1 Advance Power Electronics Research Lab, Department of Electrical Engineering, Jamia Millia Islamia, New Delhi 110025, India; kvsb272@gmail.com; 2 Department of Electrical and Computer Engineering, King Abdulaziz University, Jeddah 21589, Saudi Arabia; amalshareef1@kau.edu.sa (A.A.); amalam@kau.edu.sa (M.M.A.); 3 Computer Science Department, Faculty of Computing and Information Technology, King Abdulaziz University, Jeddah 21589, Saudi Arabia; aikhan@kau.edu.sa; 4 Center of Research Excellence in Renewable Energy (CoRE-RE), King Fahd University of Petroleum & Minerals, Dhahran 31261, Saudi Arabia; kashif.irshad@kfupm.edu.sa

**Keywords:** islanding, non-detection zone, power imbalance, voltage sag, feature extraction, support vector machine

## Abstract

This paper develops an islanding classification mechanism to overcome the problems of non-detection zones in conventional islanding detection mechanisms. This process is achieved by adapting the support vector-based data description technique with Gaussian radial basis function kernels for islanding and non-islanding events in single phase grid-connected photovoltaic (PV) systems. To overcome the non-detection zone, excess and deficit power imbalance conditions are considered for different loading conditions. These imbalances are characterized by the voltage dip scenario and were subjected to feature extraction for training with the machine learning technique. This is experimentally realized by training the machine learning classifier with different events on a 5 kW grid-connected system. Using the concept of detection and false alarm rates, the performance of the trained classifier is tested for multiple faults and power imbalance conditions. The results showed the effective operation of the classifier with a detection rate of 99.2% and a false alarm rate of 0.2%.

## 1. Introduction

The conventional power generation techniques have been under a tremendous pressure while coping with the ever-rising load demand. In addition, there are various environmental concerns in these techniques, which resulted in the need to switch towards renewable energy systems. Furthermore, with the advancement in technology, there has been a lot of innovation in the field of renewable power generation techniques, especially in the field of photovoltaics [1,2]. Moreover, the profitability of the roof top installation [3] of photovoltaic systems and their efficient interconnection with the distribution network provided a better way of handling the environmental concerns. These improvements and capabilities helped the PV system for global sustainability and to reduce the pressure of the utilities and meet the peak demand efficiently. But with the number of distributed generation (DG) systems rising, there is always a possibility of power instability [4] at the grid end, and as a result, protective schemes are required [5]. At times of power mismatch, the DGs may be unintentionally islanded from the grid [6] which may lead to a rapid loss of power, unbalanced frequency, and unregulated voltage, which can severely damage DGs. To limit the damage and avoid unintentional islanding, it is necessary that the operating state of the DGs are monitored and different faults are classified. Based on the classification, the DGs can be islanded more efficiently without causing any damage to DGs, as well as the grid. Many of the international standards, i.e., IEEE 929-1988 [7], IEC 62116 [8], and IEEE 1547-2018 [9], have come up with instructions to regulate the interconnection between DGs and utilities. These standards regulate the clearance time in case any abnormality is detected.

The islanding detection can be broadly classified into local and remote techniques [10]. During the remote detection technique, the DGs are monitored continuously by the control center with the help of methods, i.e., power line communication (PLC) [11], supervisory control, and data acquisition (SCADA) [12]. Even though the remote detection technique is reliable and efficient, it does involve substantial investment, along with its over dependence upon the communication link between DGs and the control center. If the communication link is compromised, it can lead to unsupervised operation [13]. The local detection technique can further be classified into active, passive, and hybrid techniques. In passive islanding detection [14], the components such as voltage, frequency, and harmonics are monitored for abnormality and threshold is set, hence, in case of any abnormality, the DGs are disconnected from the grid through relays. But the drawback with this method is the presence of a large non-detection zone (NDZ) [15]. Hence, to overcome the issue of NDZs, active islanding detection was introduced [15]. In case of active islanding detection, perturbation is introduced in the system and monitored for abnormality. It does present a small NDZ, but the introduction of perturbance in the system may lead to the degradation of power quality, and if it is significant, it may make the system unstable even when the system is not islanded [16]. To overcome the drawback of both the active and passive islanding technique, the hybrid islanding technique [17] was introduced, in which the perturbation is injected when abnormality is detected in the system, and as a result, the NDZ is small. However, the islanding detection time is prolonged, as both the detection methods are implemented. Many researchers have proposed different intelligent techniques [18,19] and machine learning techniques [20,21,22] in order to achieve minimum NDZs and faster islanding detection. 

Even though all the techniques presented in the literature have efficiency, issues such as the regulation of perturbance, minimal NDZs, and faster detection have not been addressed. If the load power and DG nominal power are closely matched, most of the methods are unable to detect power mismatches taking place. As a result, there is a vast scope in developing an islanding detection technique which is not just accurate but also overcomes all the underlying issues presented in the literature. 

In this paper, the support vector-based data description technique is adapted for developing an islanding classification mechanism. The proposed mechanism overcomes the problem of non-detection zones and classification accuracy by training a machine learning classifier with the data of various islanding scenarios, network outages, and power imbalance conditions. Prior to the development of the classifier, different islanding scenarios are simulated, and multiple features of the network variables are extracted. These features are based on both the system and signal characteristics and aid in efficiently discriminating the abnormalities caused by various islanding and non-islanding scenarios in the system. The novelty of the proposed methodology lies in the feature extraction process and the features identified, which help in overcoming the disadvantages of NDZs in the conventional islanding detection mechanisms. Furthermore, the detailed explanation of the adapted techniques and their importance in developing an efficient islanding classifier is discussed as follows: Section 2 discusses the operation and control of a single-phase grid-connected system. In Section 3, the possible islanding scenarios, grid requirements and non-detection zones in a grid-connected system are defined. The prerequisites and methodology for developing the classifier are discussed in Section 4, and the experiment is presented in Section 5. Section 6 provides the discussion and conclusion for the developed islanding classification approach.

## 2. Grid-Connected PV System

**Figure 1:**
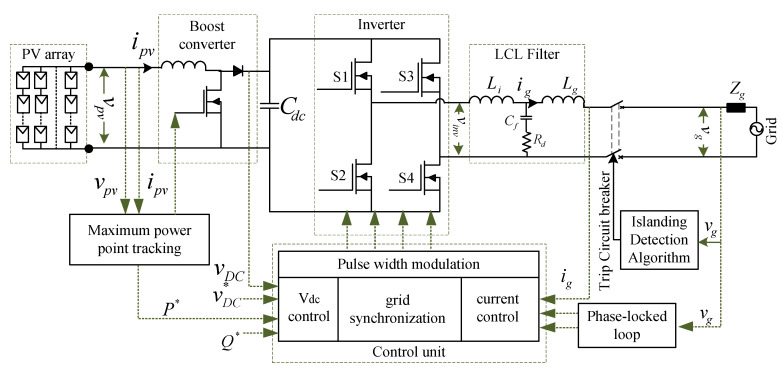
Outline of a single-phase grid-connected photovoltaic system.

The single-phase grid-connected PV systems generally deal with roof top power systems with power ranges up to 10 kW [23]. The outline of a grid-connected PV system consists of a DC–DC converter with maximum power point tracking (MPPT) control, DC/AC converter with grid tied and standalone control, filters, and other conversion technologies, as shown in Figure 1. The control of PV inverters (DC/AC converter) generally depends either on grid tied mode or standalone mode (off-grid mode). The standalone operation of a grid tied inverter deals with the control of output voltage with respect to a reference voltage, hence serving as a voltage-controlled source. However, the grid tied operation of inverters is further classified as grid forming mode, grid feeding mode, and grid supporting mode. The operation of inverters in any of these modes is dependent on the control of the output current as per the reference current values. Hence, the inverter operates as a current-controlled source in grid tied mode [24]. In both the standalone and grid-connected modes, the adapted controller must be capable of achieving reduced leakage current and should maintain current quality by limiting the harmonics due to DC current injection. In addition, the feedback controllers involved both in DC and AC operation should ensure extracting maximum power from PV panels, grid synchronization, the injection of active power in a controlled manner, and reactive power support. Further, the closed loop control of a grid tied inverter, which is a combination of inner current loop and outer voltage loop, is of utmost importance for achieving harmonic compensation and providing zero steady state errors [25]. 

### 2.1. Inner Control Loop 

The inner current loop stabilizes the filter inductor current $\left[I_{Lf}\right]$ to achieve the grid current control. Hence, the design of the filter inductor is very crucial for attenuating high frequency currents from the inverter to the grid [25] and in achieving efficient control. In general, the attenuated output current is compared with the reference current values $I_{Lf,\;peak}\times Sin\theta$ and the error generated is operated with the proportional resonant (PR) controller to vary the duty ratio. The PR controller is used to obtain a high gain $\omega_{0}$ at the grid frequency. For an ideal condition of the PR controller, the $\omega_{0}$ may increase to infinity, resulting in stability issues. Hence, a damping is considered for maintaining the non-ideal operation of the inverter. The ideal transfer function of the PR controller is given in (1) and the frequency response of damping is given in (2), as follows:

(1) $$G_{PR}\left(s\right)=K_{p}+\frac{2K_{i}s}{s^{2}+\omega_{0}}$$

(2) $$G_{PR}\left(s\right)=K_{p}\left(1+\frac{2K_{i}\times \omega_{r}s}{\left(s^{2}+\omega_{r}s+\omega_{0}^{2}\right)}\right);\;K_{i}=\frac{K_{p}}{\tau_{i}}$$

where $K_{p}$, $K_{i}$ are the gain constants, $\omega_{r}$ corresponds to resonant cutoff frequency, and $\tau_{i}$ is the time constant. The time and gain constants of the PR regulator are calculated by following the steps below [26]: 

Step 1: To calculate the gain constants, the generalized gain equation is given by:

(3) $$G\left(s\right)=G_{PR}\left(s\right)\times e^{-sT_{d}}\times G_{p}\left(s\right)$$

where $G_{p}\left(s\right)$ is the plant transfer function, and $T_{d}$ is the delay time.

Step 2: The desired phase margin $\phi_{m}$ at cross-over frequency $\omega_{c}$ is calculated from:

(4) $$G_{PR}\left(j\omega_{c}\right)\times e^{-j\omega_{c}T_{d}}\times G_{P}\left(j\omega_{c}\right)=-\pi +\phi_{m}$$

(5) $$\phi_{m}≅\omega_{c}T_{d}+\tan^{-1}\left(\tau_{i}\times \frac{\omega_{c}}{2\omega_{r}}\right)$$

Step 3: From the estimations in (5), the maximum cross-over frequency $\omega_{cmax}$ is calculated as:

(6) $$\omega_{cmax}=\frac{\left(90{}^{\circ}-\phi_{m}\right)}{T_{d}}\times \frac{\pi {}^{\circ}}{180}$$

Step 4: Furthermore, the time constant $\tau_{i}$ is calculated using (7):

(7) $$\tan^{-1}\left(\tau_{i}\times \frac{\omega_{cmax}}{2\omega_{r}}\right)=\phi_{m}-\omega_{c}T_{d}$$

Step 5: Now the gain constant $K_{p}$ is calculated by equating (1) to unity at $\omega_{cmax}$.

From the above aspects, the transfer function of the plant is given by:

(8) $$1=K_{p}\left(1+\frac{2K_{i}\times \omega_{r}\times j\omega_{cmax}}{\left({\left(j\omega_{cmax}\right)}^{2}+\omega_{r}\times j\omega_{cmax}+\omega_{0}^{2}\right)}\right)\times e^{-j}\omega_{cmax}T_{d}\times G_{p}\left(j\omega_{cmax}\right)$$

**Figure 2:**
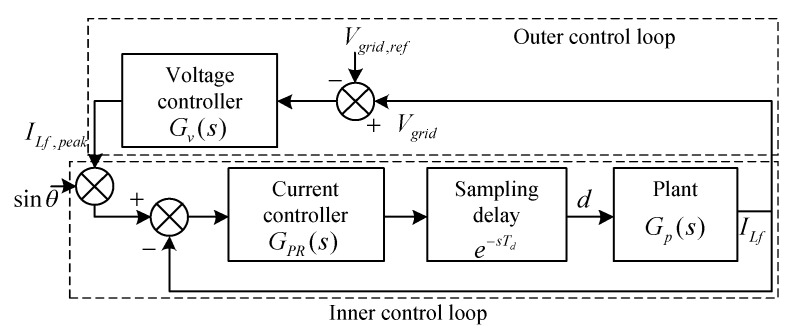
Inner current control loop and outer control loop for grid-connected inverter control.

Furthermore, the modulating output signal is compared with a carrier wave to generate gate pulses for the control of the inverter. Due to the effect of the switching cycle on the modulating output signal, a unit delay is adapted for the digital realization of the controller. This acts an inner loop compensator. The block diagram for the inner current control loop of inverter control is shown in Figure 2.

### 2.2. Outer Control Loop

The outer control loop of the inverter control deals with the generation of reference current $\left(i_{L_{f,ref}}\right)$. The block diagram for the outer control loop of the inverter is shown in Figure 2. The outer control loop has a voltage controller, which is provided with the error between the measured and reference grid voltage as input and provides the peak value of the reference output current. The transfer function for the current of filter inductor $\left(i_{L_{f}}\right)$ to the output voltage $v_{0}\left(s\right)$ is given by:

(9) $$G_{p}\left(s\right)=\frac{v_{0}\left(s\right)}{i_{L_{f}}\left(s\right)}$$

Furthermore, the design of the PR controller for the outer loop follows a similar approach for the inner current loop.

## 3. Islanding Scenarios and Grid Requirements

Most of the PV systems or distributed generation (DG) systems are usually situated near the customer loads. Like every other generating unit, these systems are prone to faults, due to short circuits and abnormal condition faults, and require electrical protection from them. Some of these faults are caused due to the influence of the grid under voltages, abnormal frequencies, unbalanced currents, and breaker operation issues during maintenance. Similarly, the DG unit faults may also impact the distribution equipment of the utility or the customer loads, resulting in power flow redistribution, increased fault currents, and over voltages. Hence, the DG protection scheme must be capable of addressing all the issues in a grid-connected environment. Considering these aspects, many national and international protection standards were recommended for the operation of DGs in grid-connected environments. Generally, each of these standards are dependent on the characteristics of the utility region and have their own specific guidelines, but few of them are universally adapted, as follows [27].

- For the protective relaying of utility consumer interconnections: IEEE C37.95-2014 [28].
- For the utility interface of PV systems: IEEE 929-2000 [29], IEC 61727 [30].
- For the interconnection of distributed energy resources with electric power interfaces: IEEE 1547-2018 [9].

**Figure 3:**
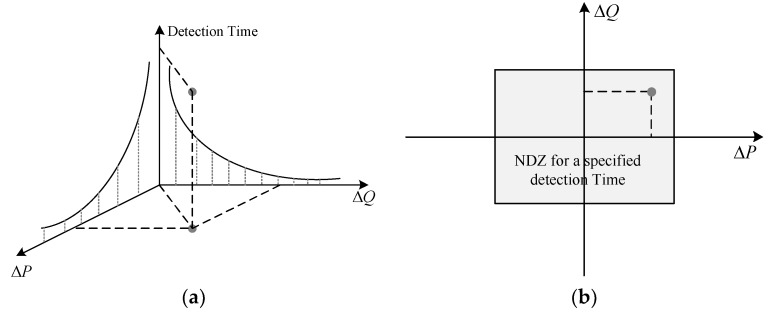
Power imbalances in a ΔP − ΔQ plane: (**a**) Power imbalance at a specified detection time, (**b**) non-detection zone due to insufficient power imbalance values [32].

One common guideline for grid interconnection is the protection of DGs from islanding. The process of DG islanding generally refers to a quick disconnection of the DG system from the utility during hazardous conditions, such as faults at utility, damage at customer equipment, and power quality degradation. This quick disconnection time generally ranges between 0 and 2 s, depending on the severity of the fault. Considering 2 s as the DG disconnection benchmark, the advancements in automatic reclosing units can cause serious damage to both utility equipment and the islanded DGs [31]. Hence, to avoid this reclosure and protect the DGs from further damage, a continuous monitoring system is required. As discussed in the literature, the active, passive, and hybrid islanding detection and monitoring techniques are widely adapted in grid-connected systems. However, these approaches operate with various drawbacks, and the limitations of the NDZ. Generally, the NDZ is characterized as a power imbalance function inside an islanded condition. This power imbalance function has two aspects: active and reactive power imbalances [32]. Any of these imbalance situations in an islanded condition can be represented as a point in a ΔP−ΔQ plane, as shown in Figure 3. Furthermore, these imbalances are characterized by the detection time associated with the operating point, as shown in Figure 3a. For a specified detection time, if the ΔP and ΔQ values are not sufficient to identify an islanding situation, those values define a non-detection zone, as shown in Figure 3b.

**Figure 4:**
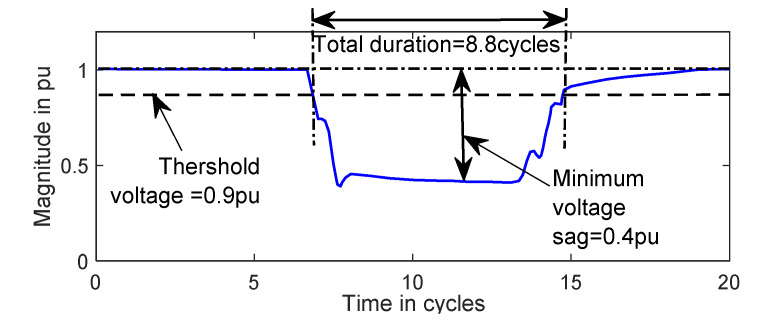
Illustration of voltage sag/dip for a threshold voltage of 0.9 pu.

Generally, the power imbalance problems are related to voltage sags or dips, temporary interruptions, frequency fluctuations, and harmonics [33]. Out of these, the voltage sags, which are characterized by a reduction in voltage magnitude for a short duration, are considered as a serious power quality issue [34,35]. As per the IEEE Std. 1159 [36], a voltage sag may range between 10% and 90% of nominal voltage for a duration of $\frac{1}{2}cycle$ to 1 min. An illustration of root mean square (RMS) voltage below the voltage dip threshold (0.9 pu) for a particular duration is shown in Figure 4. This voltage dip threshold varies depending on the purpose and region of operation [37]. Further, these voltage dips can be used for detecting islanding scenarios and overcome the phenomenon of NDZs.

## 4. Prerequisites for Classifier Development

This section discusses the development of islanding classifiers for single-phase grid-connected systems. The methodology depends on extracting multiple features from the voltage and frequency of islanding and non-islanding conditions and training them with a machine learning classifier. 

### 4.1. Data Preparation under an Islanding Scenario

A grid-connected PV system is simulated, as shown in Figure 1, using a MATLAB/Simulink environment for observing the network variables at the DG connection point. The islanding and non-islanding scenarios are implemented in the simulated system by operating the circuit breaker in the network and their impact is identified on the basis of voltage (V*)* in pu, the rate of change of voltage $\left(\frac{dv}{dt}\right)$, frequency (f), and the rate of change of frequency $\left(\frac{df}{dt}\right)$. Different islanding scenarios implemented for the purpose of developing the classifier are presented in Table 1.

**Figure 5:**
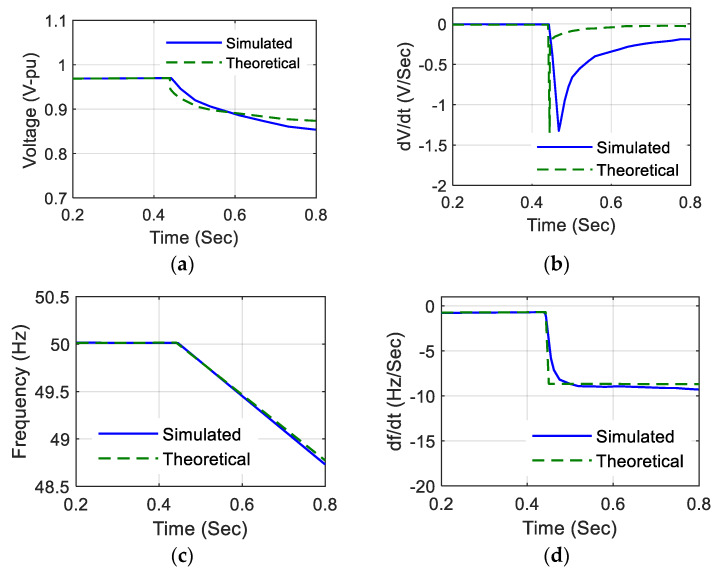
Network variables for an islanding scenario in a single-phase grid-connected system for simulation measurements and theoretical calculations: (**a**) voltage (V) in pu, (**b**) the rate of change of voltage $\left(\frac{dv}{dt}\right)$, (**c**) frequency (f), (**d**) the rate of change of frequency $\left(\frac{df}{dt}\right)$.

To achieve a reactive power imbalance in an islanding condition, a pure reactive load is added with the DG operating under a no load condition [38]. For a time-domain analysis of a PV system, an islanding condition at time t varies the network variables, as shown in Figure 5. 

The theoretical analysis of network variables under an islanding condition in Figure 5 are calculated from [21]. An active power imbalance deficit of 50% is considered between the generation and load within the islanded network. The slight difference in theoretical and simulation results is due to the action of the automatic voltage regulator equipped with the simulation model. From the above discussion, the four network variables can be obtained for different islanding scenarios to develop a classifier model. Furthermore, the obtained data are subjected to feature extraction for achieving an efficient fault classifier.

### 4.2. Feature Extraction

**Figure 6:**
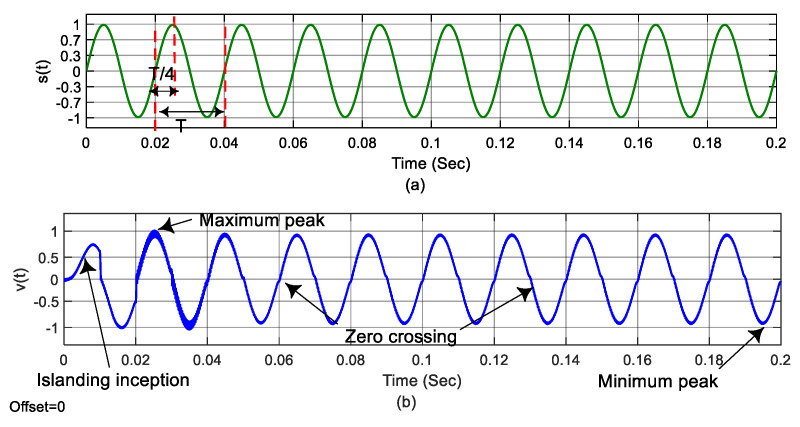
Feature extraction process: (**a**) Reference waveform s(t), (**b**) measured voltage at grid interconnection terminals v(t).

The feature extraction process transforms the raw signals into informative signatures for training with a classifier. The raw signals, normalized voltage V, the rate of change of voltage $\rho_{v}=\left(\frac{dv}{dt}\right)$, normalized frequency (f), and the rate of change of frequency $\rho_{f}=\left(\frac{df}{dt}\right)$ are obtained at the grid interconnection terminals. The rate of change of voltage and frequency are obtained from voltage and frequency variation, respectively, within a small interval of time Δt. To begin with, the feature extraction process considers a reference signal s(t) of 10 cycles for a detection time of 200 ms with a system frequency of 50 Hz, as shown in Figure 6a. Furthermore, the measured voltage signal v(t) is processed to obtain four different points, two zero crossing points, and positive and negative half cycle peaks for each cycle, as shown in Figure 6b. 

**Figure 7:**
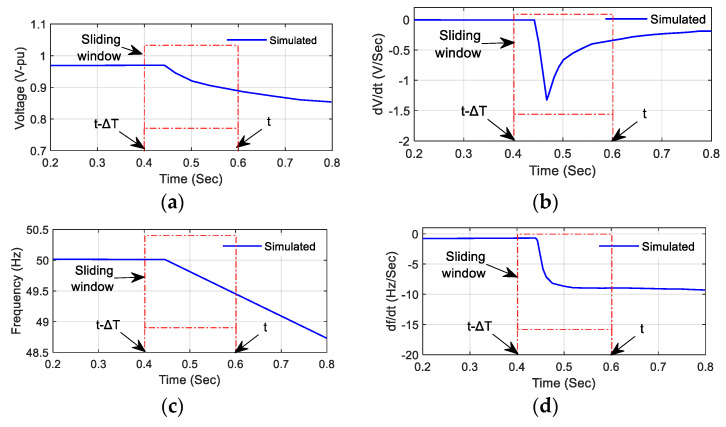
Sliding window-based feature extraction process for (**a**) voltage (V) in pu, (**b**) the rate of change of voltage $\left(\frac{dv}{dt}\right)$, (**c**) frequency (f), (**d**) the rate of change of frequency $\left(\frac{df}{dt}\right)$.

These points contribute to four different features by calculating the standard deviation inside a sliding window of width ΔT. The standard deviation feature extraction process for network variables of an islanding condition in Figure 5 are shown in Figure 7. For the condition where the active power imbalance is 50%, there is a significant variation in all the variables from the normal condition. However, at a power imbalance <10%, the variation is very low and similar to variables under a non-islanding condition [20,39]. Furthermore, the extracted features of the four variables in a sliding window are given by:

(10) $$\sigma_{V}=std\left\{v\left(\tau \right);\tau \in \left[t-\Delta T,t\right]\right\}$$

(11) $$\sigma_{f}=std\left\{f\left(\tau \right);\tau \in \left[t-\Delta T,t\right]\right\}$$

(12) $$\sigma_{\rho_{v}}=std\left\{\frac{dv\left(\tau \right)}{dt};\tau \in \left[t-\Delta T,t\right]\right\}$$

(13) $$\sigma_{\rho_{f}}=std\left\{\frac{df\left(\tau \right)}{dt};\tau \in \left[t-\Delta T,t\right]\right\}$$

where $\sigma_{v},\;\sigma_{f},\;\sigma_{\rho_{v}},$ and $\sigma_{\rho_{f}}$ correspond to standard deviation features extracted for different network variables. Furthermore, these features are represented as a feature vector for training with the classifier, as follows:

(14) $$x={\left[\sigma_{V},\;\sigma_{f},\;\sigma_{\rho_{v}},\;\sigma_{\rho_{f}}\right]}^{T}$$

### 4.3. Classifier Development

The feature vectors obtained through the feature extraction process are subjected to classifier development using a machine learning classifier. The support vector machine algorithm, which is the basic form of data descriptor techniques in machine learning, is used to develop the classifier. 

#### 4.3.1. Support Vector Machine 

For a real valued d-dimensional feature vector $\left(x_{n}\in {\mathbb{R}}^{d}\right)$ labeled as $y_{n}=\left\{-n,\ldots 0,1,2,\ldots ,n\right\}$ to indicate the class of $x_{n}$, the support vector machine (SVM) establishes a hyperplane to separate the classes. This hyperplane, or decision boundary, is defined by a scalar bias b and vector w as follows:

(15) $$g\left(x\right)=w^{T}x+b$$

In case of linearly separable data, none of the input samples $x_{n}$ satisfy the function g(x)=0. Hence, to control the separability of the data, inequalities are considered for all the data classes. Further, to generalize the classification capability of the SVM, margins are formulated during the training process by calculating the Euclidean distance between the training data and the hyperplane. The maximum Euclidean distance to the closest training samples are known as support vectors. These support vectors aid in developing an optimal hyperplane during the training process. The mathematical representation for the Euclidean distance between the training data x and the separating hyperplane is given by $\frac{\left|g\left(x\right)\right|}{\left\|w\right\|}$, where all the training data should essentially satisfy the condition

(16) $$\frac{y_{n}g\left(x_{n}\right)}{\left\|w\right\|}\geq \eta \;\forall \;n=1,2,\ldots ,N$$

where η represents the margin formulated during the training process. For a solution (w,b) and a positive scalar a, (aw,ab) is also a solution which imposes the following constraints [40]:

(17) η‖w‖=1

Therefore, from the above Equations (16) and (17), it is evident that by minimizing the Euclidean norm, an optimal separating hyperplane can be achieved, as follows:

(18) $$\underset{w}{\min}\frac{1}{2}{\left\|w\right\|}^{2}$$

(19) $$S.t\;y_{n}\left(w^{T}x_{n}+b\right)\geq 1\;\forall \;n=1,2,\ldots ,N$$

In case of linearly non-separable data, a soft margin support vector is applied and the training errors are measured through a slack variable ξ. To deal with this condition, Equations (18) and (19) are re-formulated as:

(20) $$\underset{w,\xi }{\min}\frac{1}{2}{\left\|w\right\|}^{2}+C\overset{N}{\underset{n=1}{\sum }}\xi_{n}$$

(21) $$\begin{array}{c} S.t\;y_{n}\left(w^{T}x_{n}+b\right)\geq 1-\xi_{n}\;\forall \;n=1,2,\ldots ,N\\ \xi_{n}\geq 0\;\forall \;n=1,2,\ldots ,N \end{array}$$

where C is a regularization parameter to determine the tradeoff between the minimization of the classification errors and the maximization of the margin, and $\xi_{n}$ corresponds to the slack variable measuring the training error for one misclassified sample. The square of the Euclidean norm in (18) makes the optimization problem a quadratic programming. The constraints in the quadratic programming can be solved by introducing Lagrange multipliers $\beta_{n}\geq 0$ and $\gamma_{n}\geq 0$ [40]. For a Lagrange multiplier $\beta_{n}$, the solution for the Euclidean norm is given by:

(22) $$w=\overset{N}{\underset{n=1}{\sum }}\beta_{n}y_{n}x_{n}\;\forall \;n=1,2,\ldots ,N.$$

This gives final decision boundary g(x) as:

(23) $$g\left(x\right)=\underset{\beta_{n}>0}{\sum }\beta_{n}y_{n}x_{n}^{T}x+b$$

where x corresponds to the test vector and the decision function of g(x) is given by sgn(g(x)) with a tuning parameter C.

Furthermore, in case of nonlinearly separable data, a kernel trick is used with the SVM to map the input feature vectors $x_{n}$ to high-dimensional feature space, as given by:

(24) $$z_{n}=\Phi \left(x_{n}\right)$$

This solution provides a new feature vector $z_{n}$. Generally, the kernel trick computes the inner product of original data points in mapped feature space, which forms $K\left(x_{n},x_{m}\right)=\langle \Phi \left(x_{n}\right),\Phi \left(x_{m}\right)\rangle$. This replaces $x_{n}^{T}x$ in (23) with the kernel trick to yield the decision function as follows:

(25) $$g\left(x\right)=\underset{SVs\;\mathrm{or}\;\beta_{n}>0}{\sum }\beta_{n}y_{n}K\left(x_{n},x\right)+b$$

Furthermore, to solve the optimization problem in (20) and (21), all the training samples are evaluated with the kernel. The choice of kernels for the SVM depends on the dimensionality of the data set and their description. Some of the widely used kernels, radial basis function kernel, and polynomial kernel are mathematically given, as follows [41,42]:

Gaussian radial basis function kernel:

(26) $$K\left(x,y\right)=\exp\left\{-\frac{{\left|x-y\right|}^{2}}{2\sigma^{2}}\right\}$$

Polynomial kernel:

(27) $$K\left(x,y\right)={\left(x^{T}y+1\right)}^{p}$$

where σ is similar to the regularization parameter C and p is the degree of polynomial.

#### 4.3.2. Islanding Classifier Using SVM 

The process of developing an islanding classifier is carried out in three stages: training, performance validation, and detection process.

SVM Training: According to the basics of supervised learning, the training process is carried offline, as follows:

Step 1: Perform dynamic simulations on a single-phase grid-connected PV system to generate n different islanding and non-islanding events.

Step 2: A feature vector x with four different features is obtained for every event through the feature extraction process discussed in Section 4.2. As the feature extraction process is performed for a known set of islanding and non-islanding events generated through the simulation, the obtained feature vectors are labeled a priori with the corresponding islanding or non-islanding event.

Step 3: Hence, the final feature matrix can be given by:

(28) $$F\left(n\right)={\left[\begin{array}{ccccc} x_{1} & x_{2} & x_{3} & \cdots & x_{n} \end{array}\right]}^{T}=\left[\begin{array}{cccc} \sigma_{v}\left(1\right) & \sigma_{f}\left(1\right) & \sigma_{\rho_{v}}\left(1\right) & \sigma_{\rho_{f}}\left(1\right)\\ \sigma_{v}\left(2\right) & \sigma_{f}\left(2\right) & \sigma_{\rho_{v}}\left(2\right) & \sigma_{\rho_{f}}\left(2\right)\\ \sigma_{v}\left(3\right) & \sigma_{f}\left(3\right) & \sigma_{\rho_{v}}\left(3\right) & \sigma_{\rho_{f}}\left(3\right)\\ \vdots & \vdots & \vdots & \vdots \\ \sigma_{v}\left(n\right) & \sigma_{f}\left(n\right) & \sigma_{\rho_{v}}\left(n\right) & \sigma_{\rho_{f}}\left(n\right) \end{array}\right]$$

Step 4: In this training process, the feature vector $x_{n}$ is labelled for two classes $y_{n}$, i.e., islanding ($y_{n}=-1$) and non-islanding ($y_{n}=+1$).

Step 5: Further, a k-fold cross validation is applied for the training data to conduct the classification process. Initially, the data is sampled into k subsets, and k−1 subsets are involved with the training process while the remaining subsets are used in the testing process. As the training data are nonlinear, the Gaussian radial basis function kernel is used with the soft margin SVM classification. The process is iterated for k time and the average classification accuracy is obtained to assess the performance.

Performance Evaluation: The trained SVM model yields a decision function g(x), as given in (25), which is further used for classifying the input test data as the islanding and non-islanding conditions. To assess the performance of the trained classifier on the basis of the training and testing process, the false alarm (FA) and detection rate (DR) are adapted, as shown in (29) and (30), respectively [43]. The DR corresponds to the ratio between successful and total islanding events, FA corresponds to the ratio between misclassified and total non-islanding events.

(29) $$DR=\frac{TP}{\left(TP+FN\right)}$$

(30) $$FA=\frac{FP}{\left(FP+TN\right)}$$

where TP is true positive, FN is false negative, FP is false positive, and TN is false negative. The positive and negative terms correspond to the islanding and non-islanding classification of events, respectively. Furthermores, the true positive rate deals with successful islanding classification, false negative rate deals with misclassified non-islanding conditions, the false positive rate deals with misclassified islanding conditions, and the true negative rate corresponds to successful non-islanding classifications.

**Figure 8:**
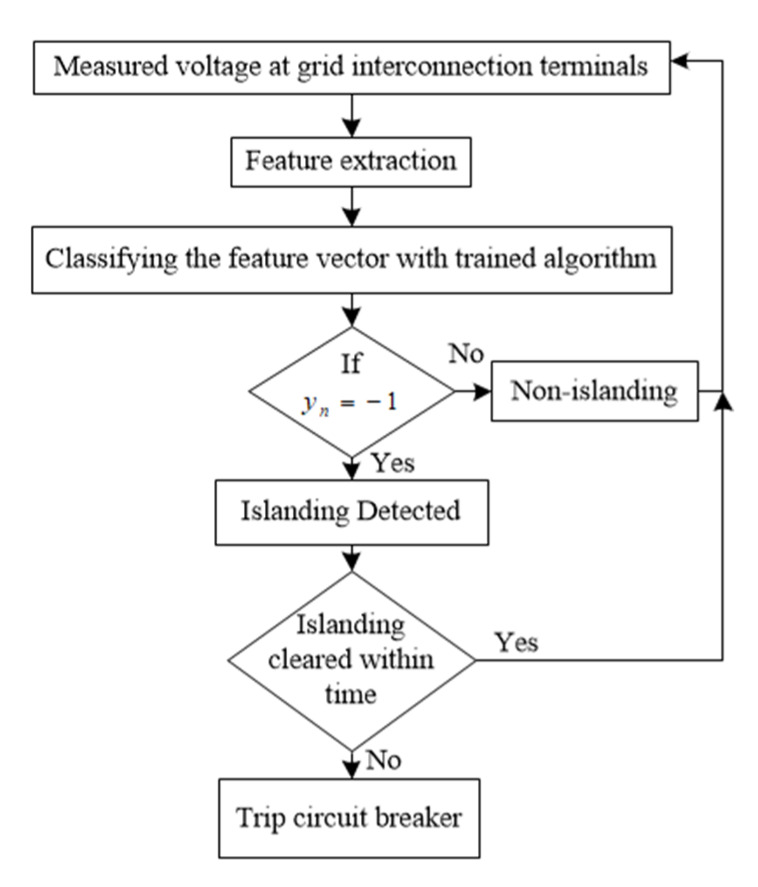
Flow diagram for islanding detection in single phase grid connected system using the trained support vector machine (SVM).

Detection Process: The islanding detection process using the trained SVM model is shown in Figure 8. The features $\left(x_{n}\right)$ of the measured data at grid interconnection are extracted and tested with the trained SVM. The extracted data are either classified as non-islanding or islanding events. In case of a non-islanding event, the process of measurement and feature extraction continues for further testing, whereas for an islanding scenario, the control parameters to stabilize the system by injecting reactive power are carried out. If the recovery process fails to stabilize the network, the islanding scenario generates a trip signal to island the PV system from the grid.

## 5. Experiment and Results 

**Figure 9:**
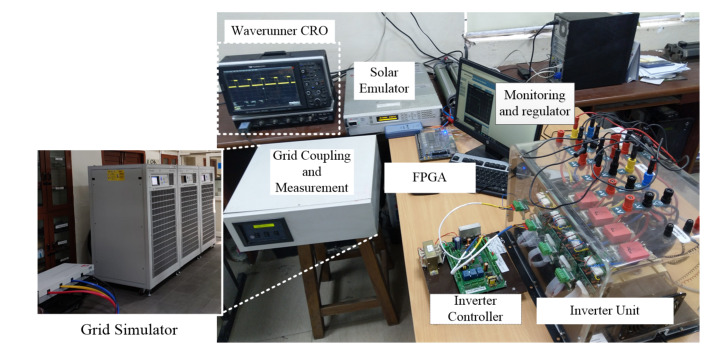
Test arrangement of a single-phase grid-connected photovoltaic system.

The developed islanding classification method is tested with a 5-kW single-phase PV system implemented using a Keysight PV simulator manufactured by Keysight Technologies India Private Limited, New Delhi, India. The PV simulator emulates the behavior of the PV arrays according to the PV module parameters and the mission profile. Furthermore, the PV simulator is connected with a single-phase inverter designed on a Semikron inverter stack manufactured by SEMIKRON Electronics Private Limited, Navi Mumbai, India. The formed single-phase PV system setup is connected to a low voltage grid, simulated using a chroma regenerative grid simulator supplied by Quantel Technologies India Private Limited, New Delhi, India. The details of the developed grid-connected photovoltaic system are given in Table 2. The trained islanding classification algorithm is dumped into an Altera Cyclone IV EP4CE115F29C7N field programmable gate array (FPGA) using a hardware description language and Quartus programming tool. The setup of the grid-connected PV system is depicted in Figure 9.

The islanding and non-islanding scenarios in the single-phase grid-connected system are created by creating fault behaviors in the chroma regenerative grid simulator. The scenarios, like load switching and shedding, the switching of capacitor banks, and the loss of lines, which do not island the DG, are considered as non-islanding scenarios. Further islanding scenarios are created by tripping the grid connection circuit breaker for different active and reactive power imbalances and creating a line to ground fault through the grid simulator software. The events generated are shown in Table 3, and all these events are experimented at 1 sec for each event and their corresponding features are extracted over a ΔT observation period. Four standard deviation features for each event are extracted for ΔT=10 cycles.

A total 990 events are generated for both islanding and non-islanding scenarios, as presented in Table 3. Furthermore, four features are extracted for each sample in both the scenarios. The total training and testing data for the events discussed in Table 3 are shown in Table 4. The SVM is trained with the above data, as discussed in Section 4.3, by setting k=5 for k-fold cross-validation, which gives an optimal regularization parameter C=300. As the data deals with real world signals and their signatures, the Gaussian radial bias function in (26) is used for mapping the nonlinear data into a high-dimensional feature space. The performance parameters of the SVM, with the adapted kernel, are shown in Table 5. Furthermore, the detection rate and false alarm rate for islanding events with different loads for the adapted kernel are given in Table 6.

**Figure 10:**
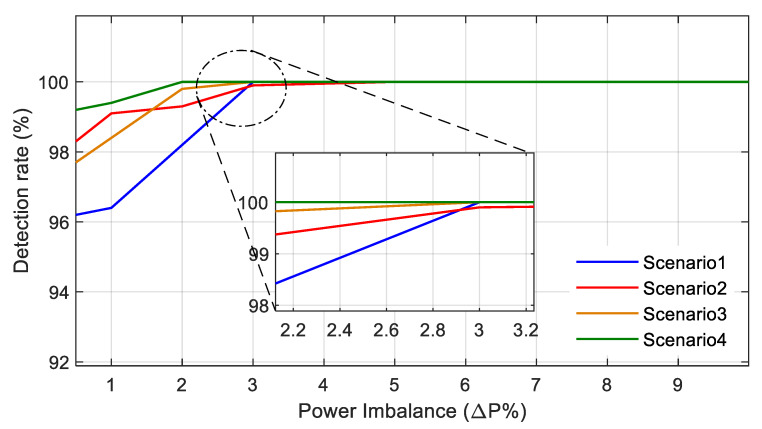
Detection rate of the classifier for power imbalance in different scenarios.

To test the performance of the developed classifier under transient conditions, which contribute to the major aspect of NDZs, different deficient power imbalances are created, ranging for active and reactive power. The power imbalance ΔP is varied between 0.5% to 10% and ΔQ is varied from 0% to 50% to test the scenarios 1–4 in Table 2. The corresponding results for the Gaussian radial basis function are shown in Figure 10 and Table 7.

The detection rate of the classifier for power imbalances in different scenarios is shown in Figure 10. For analyzing the detection accuracy along with the failure rate, the data are further represented in Table 7. The variation of the power imbalance ΔP from 0.5% to 10% simulates the impact of NDZs in the system. For power imbalances below 3% under scenario 1, the developed classifier is able to classify with an average detection rate of 96.9% and a 0% failure rate, and for power imbalances above 5%, the detection rate is 100%. Similarly, for power imbalances below 5% under scenario 2, the average detection rate is 99.15% with a 0.1% failure rate, and for power imbalances above 5%, the detection rate is 100% with a 0.1% failure rate. Furthermore, the same variation is observed for power imbalances under scenario 3 and scenario 4, and in both the conditions, the average detection rate is 98.6% and 99.3%, respectively, and the failure rate is 0.1% and 0.1%, respectively, for power imbalances below 3%. This indicates the efficiency of the developed method in identifying the islanding conditions under NDZs.

From the results, it is identified that the developed SVM classifier for islanding detection is flexible to various changes in the system configuration. The action of the proposed feature extraction method involving system characteristics (voltage and frequency), the rate of change of system characteristics, and signal characteristics (peaks and zero crossing points) has successfully aided in overcoming the problem of NDZs in the conventional islanding detection mechanisms. Furthermore, the decision boundary of the SVM obtained through different training scenarios, considering islanding, non-islanding, power imbalances, and network contingencies, is significantly able to classify any change in the circuit or network topology. By choosing the appropriate kernel trick, the classifier is able to achieve a detection rate of 99.2% and false alarm rate of 0.2%, which shows the acceptability of the method. All the above demonstrated performance analysis identifies the reliability of the developed method in terms of detection rate (accuracy) and false rate. However, while implementing the developed method with real time systems, the speed and response or detection time of the classifier, along with the associated relays, needs to be investigated. Here, the term speed depends on the data processing time for the classifier, and the detection time corresponds to the time delay associated with the time of the measurement of the data to the time of the operation of the corresponding relay. In both the cases, the classifier is expected to be quick. Hence, the detection time needs to be prioritized while developing an islanding classifier, which is identified as the future aspect of this research.

## 6. Conclusions

This paper developed an islanding classifier for the single-phase grid connected system. The developed approach involved the generating of multiple islanding and non-islanding scenarios, a feature extraction process, and a classification process. The islanding and non-islanding events are generated using a 5 kW grid-connected system. To overcome the drawback of non-detection zones in conventional islanding detection methods, the excess and deficient power imbalance conditions and their effect on the voltages of the grid-connected system are measured. Four network variables are identified for the measured voltages at the grid interconnection point. To distinguish between the variables of different events, the feature extraction process is carried out using the sliding window approach. This provides a feature vector with multiple features for each islanding event. Furthermore, the islanding events are trained with the support vector machine classifier to achieve an islanding classification mechanism. To assess the performance of the developed approach, multiple testing conditions are obtained for different loading conditions. The results of the testing approach depicted better efficiency in terms of the detection rate and lower false alarm rate to overcome the non-detection zone.

## Figures and Tables

**Table 1:** Description of different islanding scenarios considered for developing the classifier.

Islanding Type	Scenario	Description of Islanding Scenario
Islanding	Scenario 1	Tripping the circuit breaker at the grid interconnection with three different loads, constant power load, constant impedance load, and constant current load.
	Scenario 2	Creating four different power imbalance conditions with excess and deficient power: (1) Deficient active and reactive power imbalances (2) Excess active and reactive power imbalances (3) Excess active power and deficient reactive power imbalances Deficient active power and excess reactive power imbalances.
Non-Islanding	Scenario 3	Switching of inductive, capacitor and nonlinear loads for less than 10 cycles per observation for constant impedance, power, and current loads.
	Scenario 4	Transient faults with a clearing time less than 0.05 to 0.1 sec for constant impedance, power, and current loads.

**Table 2:** Hardware description for a 5 kW solar photovoltaic system.

System	Parameter	Values
PV Array Simulator 5 kWP using (Topsun solar 250 W Modules)	Module Characteristics	
	Power $\left(P_{max}\right)$	250 Wp
	Short circuit current of the module $\left(I_{sc}\right)$	14.6 A
	Open circuit voltage of module $\left(V_{oc}\right)$	22 V
	Maximum power point current $\left(I_{mpp}\right)$	13.9 A
	Maximum power point voltage $\left(V_{mpp}\right)$	18 V
Inverter Stack (Semikron)	Maximum continuous output current	600 A to 1200 $A_{RMS}$
	Switching frequency	5 kHz
	Maximum inverter output voltage	690 $V_{\mathrm{AC}}$
	DC bus voltage	1100 $V_{\mathrm{DC}}$
Grid Simulator	Output power	30 kVA
	Output voltage	0~300 V
	Output frequency	30 Hz–100 Hz

**Table 3:** Islanding events and their samples.

Event	No. of Samples	Event Class
Scenario 1	300 samples	Islanding $\left(y_{n}=-1\right)$
Scenario 2	300 samples	
Scenario 3	195 samples	Non-islanding $\left(y_{n}=+1\right)$
Scenario 4	195 samples	

**Table 4:** Division of training and testing samples for different types of loads in islanding and non-islanding scenarios.

Load	Islanding				Non-Islanding	
	Excess and Deficient Power Imbalances		Number of Samples		Number of Samples	
	ΔP(%)	ΔQ(%)	Training	Testing	Training	Testing
Constant power	0–100	0–50	60	140	60	70
Constant impedance	0–100	0–50	60	140	60	70
Constant current	0–100	0–50	60	140	60	70
Total			180	420	180	210

**Table 5:** Performance parameters of the SVM with Gaussian radial basis function.

Kernel Parameter	Value
Kernel type	Gaussian radial basis function
Kernel regularization parameter σ	1
Support vectors	36
Detection rate	99.2%
False alarm	0.2%

**Table 6:** Detection and false alarm rates for islanding classification with different loads.

Load	Detection Rate	False Alarm
Constant power	99.34%	0.34%
Constant impedance	99.6%	0.8%
Constant current	99.1%	0.4%
Constant power, current, and impedance	99.3%	0.7%

**Table 7:** Results for testing of different power imbalances with the trained SVM.

ΔP%	Islanding Conditions	Scenario 1		Scenario 2		Scenario 3		Scenario 4	
		DR(%)	FA(%)	DR(%)	FA(%)	DR(%)	FA(%)	DR(%)	FA(%)
0.5	80	96.2%	0%	98.3%	0.1%	97.7%	0.1%	99.2%	0.1%
1	80	96.4%	0%	99.1%	0.1%	98.4%	0.1%	99.4%	0.1%
2	80	98.2%	0%	99.3%	0.1%	99.8%	0.1%	100%	0.1%
3	80	100%	0%	99.9%	0.1%	100%	0.1%	100%	0.1%
5	80	100%	0%	100%	0.1%	100%	0.1%	100%	0.1%
7	80	100%	0%	100%	0.1%	100%	0.1%	100%	0.1%
10	80	100%	0%	100%	0.1%	100%	0.1%	100%	0.1%
